# Intravesical liposomal tacrolimus for hemorrhagic cystitis: a phase 2a multicenter dose-escalation study

**DOI:** 10.1007/s11255-023-03783-y

**Published:** 2023-09-19

**Authors:** Jason Hafron, Benjamin N. Breyer, Shreyas Joshi, Christopher Smith, Melissa R. Kaufman, Janet Okonski, Michael B. Chancellor

**Affiliations:** 1 Michigan Institute of Urology, Troy, MI USA; 2 University of California, San Francisco, San Francisco, CA USA; 3 Emory University, Atlanta, GA USA; 4 Baylor College of Medicine, Houston, TX USA; 5 Vanderbilt University, Nashville, TN USA; 6 Lipella Pharmaceuticals, Inc, Pittsburgh, PA USA

**Keywords:** Hemorrhagic cystitis, Topical liposomal tacrolimus, Intravesical instillation, Bladder

## Abstract

**Background:**

Hemorrhagic cystitis (HC) is an inflammatory disease of the bladder with sustained hematuria for which there is currently no approved drug treatment. We evaluated a liposomal tacrolimus preparation (LP-10) in patients with refractory moderate to severe sterile HC.

**Methods:**

This phase 2a dose-escalation study assessed the safety and efficacy of up to 2 intravesical instillations of LP-10 (2, 4, or 8 mg tacrolimus) in 13 patients with HC. Primary efficacy outcomes were changes from baseline in the number of bleeding sites on cystoscopy, microscopic urine analysis for red blood cells (RBCs), and hematuria on dipstick. Additional efficacy measures included urinary incontinence, frequency, and urgency on a 3-day diary and cystoscopy global response assessment (GRA). Blood samples for pharmacokinetic (PK) assessment were obtained in all patients.

**Results:**

Intravesical LP-10 was well tolerated, with no treatment-related severe or serious adverse events (AEs) and only 3 drug-related AEs (artificial urinary sphincter malfunction, dysuria, and bladder spasms). LP-10 blood levels showed short durations of minimal systemic uptake. Treatment resulted in significant improvements in bleeding on cystoscopy, RBC counts in urine, hematuria on dipstick, and urinary incontinence. Bleeding on cystoscopy and urinary incontinence showed dose-dependent improvements that were more pronounced in the 4 mg and 8 mg dose groups. All dose groups showed a significant improvement in cystoscopy GRA.

**Conclusion:**

LP-10 was well tolerated, with clinically relevant efficacy seen in improvements in cystoscopic bleeding, hematuria, and urinary incontinence. The benefit-risk profile supports the further clinical development of LP-10 at a tacrolimus dose of 4 mg.

## Introduction

Hemorrhagic cystitis (HC) is a diffuse inflammatory disease of the bladder with sustained hematuria and lower urinary tract symptoms (e.g., incontinence, dysuria, frequency, and urgency) in the absence of other conditions, such as active tumor, vaginal bleeding, or bleeding diathesis [1, 2].

While infectious causes of HC include bacteria, viruses, and fungi, sterile HC is typically secondary to chemotherapy or radiation [3, 4]. Chemical-induced HC is most commonly caused by chemotherapeutic drugs, such as cyclophosphamide. The bladder damage after cyclophosphamide administration has been linked with the toxic metabolite acrolein, which causes a pyroptotic reaction in the bladder urothelium with ulceration and exposure of the underlying mucosa and vasculature. Radiation-induced HC is caused by radiotherapy for urologic or pelvic malignancy [3, 4]. The reported incidence of HC ranges from below 10% to up to 35% [4], with the variability due to the wide range of potential causes of HC and differences in grading criteria used. Based on data from managed-care databases, the annual incidence of HC is estimated to be 60,000 patients in the United States [3].

The severity of hematuria in patients with HC ranges from mild and self-limiting bleeding to persistent and potentially life-threatening hemorrhage with clot retention requiring surgical intervention [5]. A frequently used grading system is that by Droller et al. [6], which classifies HC into microscopic hematuria (grade 1), macroscopic hematuria (grade 2), macroscopic hematuria with small clots (grade 3), or gross hematuria with clot requiring clot evacuation (grade 4) [6]. Vast majority of patients seeking treatment have moderate to severe HC.

Preventive measures for treatment-related HC include hyperhydration to increase urine flow, continuous bladder irrigation, and 2-mercaptoethane sulfonate to bind acrolein in urine, but outcomes have been variable [4]. Intravesical instillation of sodium hyaluronate has been used successfully to prevent and treat chemical and radiation-induced HC [3].

Treatment follows a stepwise approach, starting with conservative management and escalating to more invasive measures as required [4, 7]. In the absence of obstructive clots, oral or intravenous hydration may be sufficient. Other conservative measures include treatment of concomitant infection, cessation of anticoagulants, analgesia, and spasmolytics. Higher-grade disease requires evacuation of blood clots from the bladder before any other measures are taken. If clot evacuation is unsuccessful, cystoscopy with clot evacuation and fulguration is indicated. Persisting hematuria can be addressed with intravesical treatments, including bladder irrigation with alum, silver nitrate, prostaglandins, hyaluronic acid, or formalin, or extravesical treatments, such as hyperbaric oxygen, bladder embolization, or even cystectomy with urinary diversion [4, 7]. While conservative therapies are often ineffective, more invasive treatments have a higher risk of adverse effects. For example, prostaglandins can cause severe bladder spasms, formalin instillation may be associated with anuria, vesicle fistulae, tachycardia, and death, and surgical procedures carry a high morbidity and mortality [4].

Despite the clinical challenges of HC, there is currently no approved drug treatment for HC. Lipella Pharmaceuticals, Inc. is developing LP-10, a lipid formulated with tacrolimus, for the treatment of refractory moderate to severe sterile HC. The rationale for the use of tacrolimus in the treatment of HC is twofold. First, tacrolimus is a potent immunosuppressant that acts chiefly by inhibiting proinflammatory cytokine production following T lymphocyte activation and has a direct inhibitory effect on cell-mediated immunity [8]. It has been approved by the FDA for systemic use to prevent transplant rejection [9] and as topical ointment for moderate to severe atopic dermatitis [10]. Second, tacrolimus suppresses bleeding by causing acute arteriole vasoconstriction [11, 12].

While systemic administration carries a high risk of severe adverse events, such as nephrotoxicity and hypertension due to arterial constriction, topical administration to dermal inflammatory sites has a low incidence of mainly local and transient adverse events [13–15]. However, tacrolimus is highly lipophilic and therefore poorly soluble in water, which complicates its formulation for intravesical use [16]. These challenges can be circumvented by encapsulating tacrolimus in liposomes—spherical vesicles carrying effective payload of lipophilic drugs [17]. LP-10 was developed as a sphingomyelin liposome formulation and has been extensively tested in a nonclinical study program [17–19].

We here report the results of a phase 2a dose-escalation study to evaluate the safety and preliminary efficacy of LP-10 in patients with refractory moderate to severe sterile HC.

## Patients and methods

This was a multicenter, open-label phase 2a dose-escalation study evaluating the safety, tolerability, and efficacy of LP-10 with 2 mg, 4 mg, and 8 mg of tacrolimus in refractory moderate to severe sterile HC. The study was approved by an institutional review board and performed across 9 centers in the US after signed informed consent had been obtained.

Eligible patients were at least 18 years of age and had refractory moderate to severe sterile HC (grades 2–4) [6] for at least 3 months with at least 1 episode of hematuria with or without blood clot. Patients with intravesical therapy within 1 week before screening or clinically relevant disorders such as chronic kidney disease, congenital long QT syndrome, or bleeding diathesis, were excluded from participation.

Treatment consisted of up to 2 intravesical instillations of LP-10 in 40 mL sterile water, held in the bladder for 30 min, 3–7 days apart. Follow-up included post-treatment visits at 1 and 2 weeks and telephone contact at 4–8 weeks after the last dose. The dosages for this trial were selected based on the doses currently approved by the FDA for systemic formulations of tacrolimus [9] and the results of nonclinical studies with intravesical LP-10. Patients were sequentially assigned LP-10 doses of 2 mg, 4 mg, and or 8 mg, beginning with 2 mg. After one dose was found to be safe in four patients, the next four patients were enrolled into the next higher dose cohort.

One primary objective was to assess the overall safety of LP-10 based on standard vital signs, physical examination, blood and urine laboratory studies, blood tacrolimus levels, post-void residual volume, adverse event (AE) monitoring, and the need for blood transfusion, bladder irrigation, emergency-room visits, hospitalization, urinary catheterization, or surgery up to 2 weeks after the last dose.

The primary efficacy objective was change in hematuria from baseline to 1 and 2 weeks after the last dose based on the number of bleeding sites detected on cystoscopy, microscopic urine analysis for red blood cells (RBCs) using the high-power field (HPF) method performed by a central laboratory, and hematuria on urine dipstick.

Additional efficacy measures were the number of episodes of visible blood or blood clots in urine, urinary incontinence episodes, urinary frequency, bladder pain on a 10 cm visual analog scale (VAS), and urinary urgency on a 10 cm VAS using a 3-day patient diary to be completed before each study visit, a 7-point cystoscopy global response assessment (GRA), the Urogenital Distress Inventory (UDI-6) [20], and health-related quality of life using the EQ-5D-3L [21].

Blood samples for pharmacokinetic (PK) assessment were obtained in all patients at each LP-10 dose level before and 1, 2, 4, 48, and 72 h after the first dose or until blood tacrolimus levels were non-detectable.

Treatment was halted in case of unacceptably high levels of tacrolimus of > 15 ng/mL in blood or adverse events, such as concurrent or suspected urinary tract infection, persistent elevated blood pressure (> 160/90 mmHg), intolerable pain, clinically significant urinary retention, or allergic reactions.

*Statistical methods*: All interval (or continuous) outcomes were summarized parametrically (via mean and standard deviation) and, upon confirmation of normality, the Student’s *t* test was used for evaluating statistical significance. All ordinal outcome data were summarized and evaluated using non-parametric tests. The significance level for all hypothesis testing was α = 0.20 as early-phase trials, especially orphan diseases, involve a limited participant pool and a marginally increased risk of Type I errors could be tolerable to avoid overlooking possible treatment benefits.

The paired binomial test was used when comparing treatment to baseline for ordinal measures. Pairing was achieved by evaluating the response to therapy on a per-subject basis. Each paired measurement was classified into one of five ordinal categories: much better, better, same, worse, and much worse, or three categories: better, same, and worse. Distinctions between better and much better corresponded to whether the response was a one or more-than-one category improvement, and the distinction between worse and much worse was applied analogously. Where applicable, the binomial distribution test-statistic was generalized to the multi-nomial distribution. The Mann–Whitney *U* test was used when evaluating evidence of a dose response. The paired derivative outcomes for the two groups being compared (i.e. the response to treatment of the 4 mg dose group to that of the 2 mg dose group) were ranked for the evaluation of the *U*-statistic. In such calculations the 2 mg dose group was treated as the control (in lieu of a placebo control).

## Results

Between February 2021 and October 2022, a total of 15 patients (1 female, 14 males) were screened, and 13 patients were enrolled sequentially into the 2 mg, 4 mg, and 8 mg dose cohorts and completed the trial. Demographic and clinical variables are shown in Table 1. All patients were male, and median age in the 2 mg, 4 mg, and 8 mg dose cohorts was 77, 72, and 48.5 years, respectively. The most frequent prior therapies for HC were hyperbaric oxygen therapy in six patients (46%) and bladder fulguration in five patients (38%). Three of the 13 patients (23%) received only one dose of LP-10 because they felt well after the first dose and chose to not receive a second dose, so that a total of 23 instillations were administered.

**Table 1:** Patient characteristics and number of LP-10 instillations, by dose level

	LP-10 2 mg (*n* = 4)	LP-10 4 mg (*n* = 4)	LP-10 8 mg (*n* = 5)
Age in years, median (range)	77 (65–89)	72 (63–81)	48.5 (25–72)
Gender			
Male	4 (100%)	4 (100%)	5 (100%)
Female	0 (0%)	0 (0%)	0 (0%)
Pelvic cancer			
Prostate	4 (100%)	4 (100%)	1 (20%)
Bladder	–	–	2 (40%)
Lymphoma	–	–	2 (40%)
Cancer treatment			
Prostatectomy	4 (100%)	2 (75%)	2 (40%)
External beam radiotherapy	3 (75%)	2 (50%)	2 (40%)
Radiotherapy	1 (25%)	2 (50%)	2 (40%)
Chemotherapy	–	–	2 (40%)
Androgen deprivation therapy	–	1 (25%)	1 (20%)
Prior therapies for HC			
Hyperbaric oxygen therapy	2 (50%)	4 (100%)	–
Bladder fulguration	1 (25%)	1 (25%)	3 (60%)
Bladder irrigation	–	–	1 (20%)
Imipramine	1 (25%)	–	–
Antimuscarinic	–	–	1 (20%)
Tamsulosin	–	–	1 (20%)
Current HC symptoms			
Hematuria	3 (75%)	4 (100%)	4 (80%)
Blood clots in urine	2 (50%)	1 (25%)	2 (40%)
Incontinence	1 (25%)	1 (25%)	–
Urinary frequency	1 (25%)	1 (25%)	–
Urinary urgency	–	–	1 (20%)
Bladder spasms	–	1 (25%)	–
Lower abdominal pain	1 (25%)	–	–
LP-10 instillations			
1 instillation	0	2 (50%)	1 (20%)
2 instillations	4 (100%)	2 (50%)	4 (80%)

*HC* hemorrhagic cystitis

Data are given as number of patients (%), unless otherwise noted

### Number of bleeding sites on cystoscopy

**Fig. 1:**
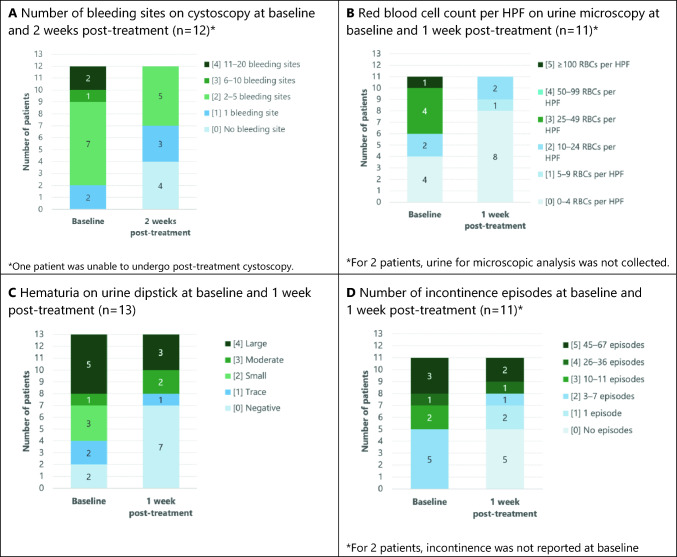
Clinical outcomes of intravesical instillation of LP-10 at doses of 2 mg, 4 mg, and 8 mg at baseline and post-treatment, all dose levels

The number of patients without bleeding sites on cystoscopy increased from 0 patients at baseline to 4 patients 2 weeks post-treatment, and the number of patients with 1 bleeding site increased from 2 to 3. None of the remaining patients had more than 2–5 bleeding sites, with the 2 patients with 11–20 bleeding sites at baseline having improved to 1 and 2–5 bleedings sites, respectively (Fig. 1A).

At 2 weeks, all dose groups showed a statistically significant improvement from baseline in the number of bleeding sites, with p values in the 2 mg, 4 mg, and 8 mg dose groups of 0.16, 0.06, and 0.04, respectively. This dose–response relationship was confirmed when outcomes were compared between dose groups. Thus, the 4 mg dose showed a trend towards a significant improvement over the 2 mg dose group (*p* = 0.26), and the 8 mg dose group showed a statistically significant improvement over the 2 mg dose group (*p* = 0.20). Treatment response did not improve in a clinically meaningful way when increasing the dose from 4 to 8 mg (*p* = 0.5) (Table 2).

**Table 2:** Number of bleeding sites on cystoscopy at baseline and 2 weeks post-treatment, by dose level

Number of bleedings sites at baseline and 2 weeks post-treatment							
Severity rank	Number of bleeding sites	LP-10 2 mg (*n* = 3)^a^		LP-10 4 mg (*n* = 4)		LP-10 8 mg (*n* = 5)	
		Baseline	2 weeks	Baseline	2 weeks	Baseline	2 weeks
4	11–20	–	–	–	–	2 (40%)	–
3	6–10	1 (33%)	–	–	–	–	–
2	2–5	2 (67%)	2 (67%)	4 (100%)	1 (25%)	1 (20%)	2 (40%)
1	1	–	1 (33%)	–	1 (25%)	2 (40%)	1 (20%)
0	0	–	–	–	2 (50%)	–	2 (40%)
*p* value for change^b^		0.16		0.06		0.04	

Change in the number of bleeding sites between baseline and 2 weeks			
	LP-10 2 mg (*n* = 3)^a^	LP-10 4 mg (*n* = 4)	LP-10 8 mg (*n* = 5)
Much worse	–	–	–
Worse	–	–	1 (20%)
No change	1 (33%)	1 (25%)	–
Better	2 (67%)	1 (25%)	1 (20%)
Much better	–	2 (50%)	3 (60%)
*p* value for comparison with 2 mg tacrolimus^c^		0.26	0.20
*p* value for comparison with 4 mg tacrolimus^c^			0.50

Better/worse = change to adjacent severity rank, much better/much worse = change by ≥ 2 severity ranks

^a^One patient was unable to undergo post-treatment cystoscopy

^b^Paired binomial test

^c^Wilcoxon Mann–Whitney test

### Red blood cell count on urine microscopy

The number of patients with 0–4 urinary RBCs per HPF on microscopy doubled from 4 at baseline to 8 at 1 week post-treatment, and none of the remaining 3 patients exhibited more than 10–24 RBCs per HPF (Fig. 1B). At 1 week, all dose groups showed an improvement in the number of RBCs per HPF on urine microscopy compared to baseline, which was statistically significant in the 2 mg and 4 mg dose groups (Table 3).

**Table 3:** Red blood cell count per high-power field on urine microscopy at baseline and 1 week post-treatment, by dose level

Severity rank	RBC count per HPF	LP-10 2 mg (*n* = 2)^a^		LP-10 4 mg (*n* = 4)		LP-10 8 mg (*n* = 5)	
		Baseline	1 week	Baseline	1 week	Baseline	1 week
5	≥ 100	–	–	–	–	1 (20%)	–
4	50–99	–	–	–	–	–	–
3	25–49	1 (50%)	–	2 (50%)	–	1 (20%)	–
2	10–24	1 (50%)	–	1 (25%)	1 (25%)	–	1 (20%)
1	5–9	–	–	–	–	–	1 (20%)
0	0–4	–	2 (100%)	1 (25%)	3 (75%)	3 (60%)	3 (60%)
*p* value for change^b^		0.12		0.11		0.32	

^a^For 2 patients, urine for microscopic analysis was not collected

^b^Paired binomial test

### Hematuria on urine dipstick

The number of patients negative for hematuria on urine dipstick increased from 2 at baseline to 7 post-treatment (Fig. 1C). At 1 week, all dose groups demonstrated an improvement in urine dipstick measurement for blood, which was statistically significant in the 2 mg and 4 mg dose groups (Table 4).

**Table 4:** Hematuria on urine dipstick at baseline and 1 week post-treatment, by dose level

Severity rank	Severity of hematuria on dipstick	LP-10 2 mg (*n* = 4)		LP-10 4 mg (*n* = 4)		LP-10 8 mg (*n* = 5)	
		Baseline	1 week	Baseline	1 week	Baseline	1 week
4	Large	1 (25%)	1 (25%)	2 (50%)	1 (25%)	2 (40%)	2 (40%)
3	Moderate	–	–	1 (25%)	1 (25%)	–	–
2	Small	1 (25%)	–	1 (25%)	–	1 (20%)	–
1	Trace	1 (25%)	–	–	1 (25%)	1 (20%)	–
0	Negative	1 (25%)	3 (75%)	–	1 (25%)	1 (20%)	3 (60%)
*p* value for change*		0.20		0.06		0.32	

*Paired binomial test

### Number of incontinence episodes

The 4 mg and 8 mg dose groups demonstrated a statistically significant improvement over baseline in the number of incontinence episodes per 3-day period, a continuous measure that clustered into 6 severity categories. Compared with the 2 mg dose group, the 4 mg and 8 mg dose groups showed a dose–response relationship with respect to the number of incontinence episodes documented (Fig. 1D). The 4 mg dose group showed improvement over the 2 mg group (*p* = 0.20), and the 8 mg dose group showed improvement over the 2 mg group (*p* = 0.11). Treatment response did not improve in a clinically meaningful way when increasing the dose from 4 to 8 mg (Table 5).

**Table 5:** Number of incontinence episodes using the 3-day patient diary at baseline and 1 week post-treatment, by dose level

Number of incontinence episodes at baseline and 1 week post-treatment							
		Baseline	1 week	Baseline	1 week	Baseline	1 week
5	45–67	1 (33%)	1 (33%)	2 (50%)	1 (25%)	–	–
4	26–36	–	1 (33%)	–	–	1 (25%)	–
3	10–11	1 (33%)	–	–	–	1 (25%)	–
2	3–7	1( 33%)	–	2 (50%)	–	2 (50%)	1 (25%)
1	1	–	–	–	1 (25%)	–	1 (25%)
0	0	–	1 (33%)	–	2 (50%)	–	2 (50%)
*p* value for change^b^		0.58		0.20		0.11	

Change in the severity of incontinence between baseline and 1 weeks post-treatment			
	LP-10 2 mg (*n* = 3)	LP-10 4 mg (*n* = 4)	LP-10 8 mg (*n* = 4)
Worse	1 (33%)	–	–
No change	1 (33%)	1 (25%)	–
Better	1 (33%)	3 (75%)	4 (100%)
*p* value for comparison with 2 mg tacrolimus^c^		0.20	0.11
*p* value for comparison with 4 mg tacrolimus^c^			0.34

Better/worse = change to adjacent severity rank, much better/much worse = change by ≥ 2 severity ranks

^a^For 2 patients, incontinence was not reported on 3-day diary at baseline

^b^Paired binomial test

^c^Wilcoxon Mann–Whitney test

### Other efficacy endpoints

All dose groups demonstrated a statistically significant improvement in the investigator-reported cystoscopy GRA between baseline and 2 weeks (*p* = 0.16 in the 2 mg dose group, *p* = 0.09 in the 4 mg dose group, and *p* = 0.01 in the 8 mg dose group). No statistically significant differences between baseline and post-treatment were seen in the number of episodes of visible blood or blood clots in urine, urinary frequency, bladder pain VAS, or urinary urgency VAS as assessed using the 3-day patient diary, or in the UDI-6 and EQ-5D-3L.

### Safety

Six of the 13 enrolled patients (46%) experienced 12 AEs (Table 6), all of which resolved within a few hours to days after occurrence. Most AEs were mild in severity, and there were no drug-related serious AEs. Drug-related AEs occurred in 3 patients (26%) (i.e., artificial urinary sphincter malfunction, dysuria, and bladder spasms). In terms of instillation-related AEs, urinary tract infection, dysuria, bladder spasms, and urinary urgency were reported.

**Table 6:** Summary of adverse events

	Severity	LP-10 2 mg (*n* = 4)	LP-10 4 mg (*n* = 4)	LP-10 8 mg (*n* = 5)	Total
Total number of AEs		4	2	6	12
Patients with at least 1 AE		3	1	2	6
Headache	Mild	1 (25%)			1
Arthralgia	Mild	1 (25%)			1
Urinary tract infection	Mild	1 (25%)^c^			1
Artificial urinary sphincter malfunction	Mild	1 (25%)^b^			1
Dysuria	Mild		1 (25%)^b,c^	1 (20%)^c^	2
Urinary retention (due to bladder blood clot)	Severe		1 (25%)		1
Hypotension	Severe^a^			1 (20%)	1
Weakness and dizziness	Severe^a^			1 (20%)	1
Bladder spasms	Moderate			1 (20%)^b,c^	1
Flank pain	Mild			1 (20%)^c^	1
Urinary urgency	Mild			1 (20%)^c^	1

^a^Serious adverse event (SAE). Both SAEs occurred in the same patient before LP-10 instillation, and neither worsened during or after instillation

^b^Drug-related

^c^Instillation-related

The frequency of AEs was lowest in the 4 mg group, with 1 patient reporting 2 AEs, i.e., dysuria and urinary retention due to a bladder blood clot. The patient’s mild dysuria was considered related to LP-10 and instillation, whereas the severe urinary retention, which occurred 4 weeks after dose 2, was considered unrelated to either LP-10 or instillation, with the patient’s baseline and post-treatment post-void residual volumes 0 mL.

All tacrolimus whole blood levels obtained were below the upper safety limit of 15 ng/mL. Blood levels after administration of 2 mg and 4 mg tacrolimus peaked at 1 h and were undetectable by 4 h. In the 8 mg dose group, 4 of 5 blood tacrolimus levels peaked at 1 h, and levels were undetectable in 3 of 5 patients by 4 h and in all patients by 48 h. At no time point did blood tacrolimus levels reach 50% of the upper safety limit of 15 ng/mL. The highest blood tacrolimus level was 7.3 ng/mL at 1 h in 1 patient in the 8 mg dose group, which decreased to 3.8 ng/mL at 2 h and was undetectable at 4 h.

Blood creatinine levels remained within the normal range in all patients. There were no significant changes in vital signs and no increases in post-void residual urine volume in any patient throughout the study.

## Discussion

In this dose-ranging phase 2a study in 13 patients with sterile refractory HC, intravesical treatment with LP-10 was found to be safe and well tolerated, with no treatment-related severe or serious AEs and only 3 drug-related AEs that were transient in nature. The frequency of AEs was lowest in the 4 mg group. The analysis of tacrolimus blood levels demonstrated a short duration of systemic uptake, with tacrolimus levels below the safety threshold of 15 ng/mL in all patients and undetectable by 4 h in 85% of patients. These results are in line with those obtained in PK studies with topical tacrolimus in patients with atopic dermatitis, in which peak blood concentrations ranged from undetectable to 20 ng/mL after single or multiple doses of 0.03% and 0.1% tacrolimus ointment [10]. By comparison, mean peak tacrolimus blood levels after oral administration at a dose of 0.3 mg/kg/day in kidney and liver transplant recipients were 24.2 and 68.5 ng/mL, respectively [9]. The low and transient blood tacrolimus levels after intravesical administration of LP-10 in this study support its localized action in the bladder with minimal systemic exposure.

Tacrolimus is a potent macrolide immunosuppressant that acts by inhibiting calcineurin, thereby inhibiting T cell proliferation and the production of a range of inflammatory cytokines [8]. Topical tacrolimus was first approved by the FDA in 2000 for the noncontinuous treatment of moderate to severe atopic dermatitis. However, it has also been investigated and found to be effective in other disorders, including psoriasis [22–25], vitiligo [26–28], oral lichen planus [29, 30], inflammatory eye disorders [31, 32], or inflammatory bowel disease [33, 34].

A key benefit of topical tacrolimus is that its side effects are limited to local immune response mechanisms and that severe adverse reactions seen with systemic administration are rare. Even with long-term use in atopic dermatitis, topical tacrolimus causes mainly local and transient symptoms, such as burning, stinging, soreness, or itching [10, 15].

The good tolerability of local tacrolimus prompted us to investigate its potential in modulating the immune response underlying sterile HC by intravesical delivery to the bladder urothelium, i.e., the primary site of tissue damage in cystitis [35]. While the delivery of tacrolimus is limited by its hydrophobic nature, it becomes highly bioavailable when formulated with hydrophilic substances such as liposomes [16]. We have previously shown, in PK studies in the rat, that the use of liposomes as a delivery vehicle increases endocytosis while decreasing systemic exposure and vehicle-related toxicity compared with non-encapsulated tacrolimus [17, 18].

We also demonstrated, in the cyclophosphamide-induced HC rat model, that instillation of liposomal tacrolimus into the bladder reversed cyclophosphamide-induced bladder hyperactivity by suppressing the local inflammatory reaction as indicated by a decrease in the number of inflammatory cells and suppression of edema. Liposomal tacrolimus also reversed the cyclophosphamide-induced EP4 receptor upregulation in the bladder as well as increases in urine PGE2 and bladder tissue PGE2 and IL-2 levels [17].

In a radiation-induced HC model in the rat, we found that intravesical liposomal tacrolimus resulted in a dose-dependent decrease in micturition intervals, an indicator of bladder function, without inducing short-term skin or gastrointestinal damage [19].

The results of the nonclinical studies with liposomal tacrolimus are mirrored by the efficacy signals obtained in this clinical study. Treatment with LP-10 resulted in significant improvements in the number of bleeding sites on cystoscopy, RBC counts on urine microscopy measured by a central laboratory, hematuria on dipstick, and urinary incontinence. Moreover, two efficacy outcome measures, i.e., the number of bleeding sites on cystoscopy and urinary incontinence, showed a dose-dependent response, with improvements in the higher-dose groups (4 mg and 8 mg) more pronounced than in the low-dose group (2 mg). These improvements in objectively measured bleeding severity and patient-reported urinary incontinence were complemented by significant improvements in the investigator-reported cystoscopic GRA in all dose groups.

The primary focus of this phase 2a dose-escalation study was to establish the most promising dose for the future development of LP-10 in the treatment of moderate to severe HC and to evaluate its initial clinical safety profile. The most favorable benefit-risk profile in this study was seen in the 4 mg group, supporting the further clinical development of LP-10 at a tacrolimus dose of 4 mg.

Limitations of our study include the small sample size and the absence of a control group. Despite their preliminary nature, all efficacy results point in the same positive direction and derive from a combination of objectively measured, patient-reported, and investigator-reported measures.

Of the objective outcome measures in this study, urine microscopy and urine dipstick blood measurements are prone to false-positive and false-negative results and are also subject to variability associated with the transitory nature of urothelial bleeding and urine production. Therefore, direct observation of bleeding sites by cystoscopy may be the most efficient efficacy indicator for measuring regression of HC. In the current study, 58% of patients demonstrated complete or near-complete resolution of bleeding on cystoscopy.

## Conclusion

LP-10 was found to be safe and well tolerated in patients with refractory moderate to severe sterile HC, with very short durations of minimal systemic uptake of tacrolimus. Clinically relevant efficacy was seen in terms of improvements in cystoscopic bleeding, hematuria, and patient-reported urinary incontinence. A dose–response relationship was evident for the reduction in cystoscopic bleeding and the decrease in patient-reported urinary incontinence. The clinical benefit-risk profile seen in this study supports the further clinical development of LP-10 at a tacrolimus dose of 4 mg.

## Data Availability

The datasets generated and analyzed during the phase 2a study by Lipella Pharmaceuticals are not publicly available due to privacy and confidentiality concerns. However, de-identified summary data can be made available upon request.
